# Awareness Regarding Sex Hormone Disruptors in Everyday Products Among Females of Reproductive Age in Al-Jouf, the Kingdom of Saudi Arabia

**DOI:** 10.7759/cureus.34255

**Published:** 2023-01-27

**Authors:** Majd F Alkhalidi, Rahaf H Alruwaili, Araa G Alruwaili, Nouf F Alqunayfith, Badur M Alaried, Raghad A Albader, Manal E Telb

**Affiliations:** 1 Medicine and Surgery, College of Medicine, Jouf University, Sakaka, SAU; 2 Medicine and Surgery, Collage of Medicine, Jouf University, Sakaka, SAU; 3 Medical Biochemistry and Molecular Biology, College of Medicine, Jouf University, Sakaka, SAU

**Keywords:** knowledge, awareness, females, sex hormones, endocrine disrupter chemicals

## Abstract

Background and objective

Endocrine-disrupting chemicals (EDCs) are natural or synthetic molecules that can alter and affect the operations of the hormonal system of an organism. These compounds include plastic consumer products and food containers such as phytoestrogen, which is also naturally present in food. EDCs can be found in the cord blood and maternal blood of pregnant women, as well as colostrum. Hence, they may affect not only the mother but also the offspring. In this study, we aimed to evaluate the awareness among females of reproductive age regarding the nature, source, as well as physiological and psychological burden associated with sex hormones disruptors.

Methods

A descriptive cross-sectional study was conducted among females between the age of 15-45 years in the Al-Jouf region, Saudi Arabia. A self-administrated questionnaire was used as the data collection tool; it consisted of multiple-choice questions to obtain information on the awareness among the females. In this study, females were classified into those with good knowledge and those with poor knowledge based on their level of knowledge by using a scoring system with a total score of 12. IBM SPSS Statistics version 24 (IBM Corp., Armonk, NY) was used to analyze the collected data.

Results

The study included 491 females; 6.6% of them had been using soya-containing products for a long time, and 32.5% reported using oatmeal for a long time. The majority (86.2%) did not use any other hormonal therapy. There were significant differences in the knowledge about sex hormone disruptors among the participants, and women with poorer knowledge about sex hormone disruptors were significantly less likely to report the long-time usage of soya-containing food when compared to women with greater knowledge (2.2% vs. 4.2%, p<0.001). The results showed that women with poorer knowledge were also significantly less likely to report the usage of hormonal therapies when compared to women with greater knowledge (6.7% vs. 7.2%, p<0.001), indicating that the usage of these chemicals is higher in women with greater knowledge although they are aware of their effects.

Conclusion

The study showed that females had good knowledge about the nature and usage of EDCs but poor knowledge about their impact. The knowledge of females was associated with their behavior regarding the usage of such products.

## Introduction

Endocrine-disrupting chemicals (EDCs) are defined as chemical substances that can mimic or interfere with the normal functions of the endocrine system and hormone biosynthesis, resulting in a deviation of normal hormonal control in the normal cells. These deviations are linked with developmental and reproductive health problems, especially in females. These chemical disruptors are found in food, cosmetics, and many other daily products [1]. Several toxic industrial chemicals tend to be EDCs for humans and are detected in human body fluids. They have been associated overwhelmingly with various endocrinological and reproductive impairments [2]. Among these are xenoestrogens, which are estrogen-like compounds. They are mostly found in plastic wares, personal care products, and pesticides [3]. Another group of these xenoestrogens is phytoestrogens from edible plant sources, most notably soybean, and sesame flaxseeds among others, as well as grains, oil, and other products. Xenoestrogens can bind to estrogen receptors in the human body, leading to detrimental effects. This has an adverse consequence for estrogen-dependent health outcomes, which include reproductive health, puberty, and pregnancy [4]. A less commonly known xenoestrogen, triclosan (5-chloro-2-(2,4-dichloro phenoxy) phenol), is a broad-spectrum antibacterial, mostly used in cosmetics, soaps, and other consumer products. The detection of triclosan in human breast milk, urine, and serum due to its extensive use has raised concerns due to its association with numerous adverse health outcomes, including cancer development [5]. Given the adverse effects of EDCs on female reproductive health, fertility, and well-being, our research aimed to assess the awareness regarding sex hormone disruptors in everyday products among women in Al-Jouf, Saudi Arabia, and to enhance the understanding of these factors affecting their fertility in order to help females make healthy choices and improve their reproductive health. To that end, we conducted a pre-validated questionnaire-based study in 2021-2022, with the following objectives in mind: (1) to evaluate the knowledge about the nature, sources, and physiological and psychological burden of sex hormones disruptors among females of reproductive age, and (2) to analyze the relationship between awareness about the impact of exposure to sex hormones disruptors and sociodemographic characteristics.

## Materials and methods

Study design, setting, and sampling method

This was a descriptive cross-sectional study conducted among women of reproductive age in the Al-Jouf region, Saudi Arabia. A pre-validated form was circulated online through social media; the sampling method used was convenience sampling.

Sample size

The population of the Al-Jouf region is 531,952 according to the General Authority for Statistics (GAStat) [6]. We targeted a female sample between the ages of 15 to 45 years, and it amounted to 127,104, which represents 23% of the total population. We calculated the sample size by keeping the confidence interval at 95% and the margin of error at 5%, and the sample size determined was 383. But we collected data from 491 people to account for any potential missing or incomplete data.

Tool for data collection

Self-administered questionnaires consisting of multiple-choice questions were used. The questionnaire was designed to obtain information about the usage as well as the awareness regarding endocrine disruptors, such as the long-time consumption of soya-containing foods (six months and above). In addition, we obtained data on variables such as sociodemographic status including age, educational status, weight, place of residence, and income. Also, we obtained data on other variables regarding the reproductive status such as menarche, duration of the menstrual cycle, and its regularity. We classified the participants into two categories based on a scoring system with a total score of 12: a score of 0-6 indicated poor knowledge while that of 7-12 showed good knowledge. The questionnaire has been partly adapted from the study by Anjum et al. about knowledge and attitude toward xenoestrogens [7], and some questions were added to it. The questionnaire was validated through a pilot study.

Inclusion and exclusion criteria

The inclusion criteria were as follows: females of reproductive age (15-45 years) residing in the Al-Jouf region. The exclusion criteria were as follows: incomplete forms and non-Arabic speakers.

Ethical consideration 

The study was conducted after obtaining the approval of the Ethics Review Committee (LCBE) of Al-Jouf University (approval no: 6-03-43). No identifying data were included in the questionnaire.

Statistical analysis 

The data were analyzed by using IBM SPSS Statistics version 24 (IBM Corp., Armonk, NY). Descriptive statistics and a comparison of the variables were carried out.

## Results

A total of 491 participants were involved in this study. All of them were asked about certain products that can lead to hormonal disruption. Our findings showed that the percentage of participants who were using soya-containing products for a long period was 6.6%. Among the total participants, 32.5% used oatmeal and 33.6% were using contraceptive pills. The proportion of participants who were unsure if they used soya-containing products was 21.8% (Table 1).

**Table 1:** Utilization of different products

		N	%
Did you use soya-containing products for a long period?	No	352	71.7%
	Not sure	107	21.8%
	Yes	32	6.6%
Did you use any of the following products for a long period?	Lavender/jasmine oils	1	0.2%
	Flax seeds	19	4.4%
	Soya seed or its derivatives	7	1.6%
	Oatmeal	141	32.5%
	I did not use any	266	61.3%
Have you ever used contraception pills?	Yes	165	33.6%
	No	326	66.4%
Have you ever used any other hormonal therapy?	Yes, for a long period	25	5.1%
	Yes, for a short period	43	8.8%
	No	423	86.2%

**Figure 1:**
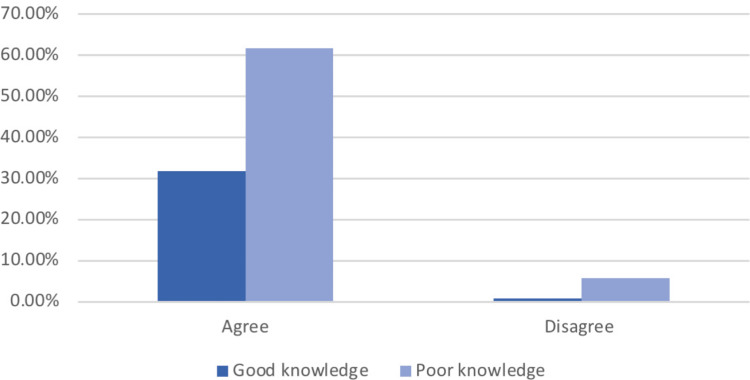
Knowledge regarding EDCs' nature and the level of agreement about their effects on period duration and intensity EDC: endocrine-disrupting chemical

**Figure 2:**
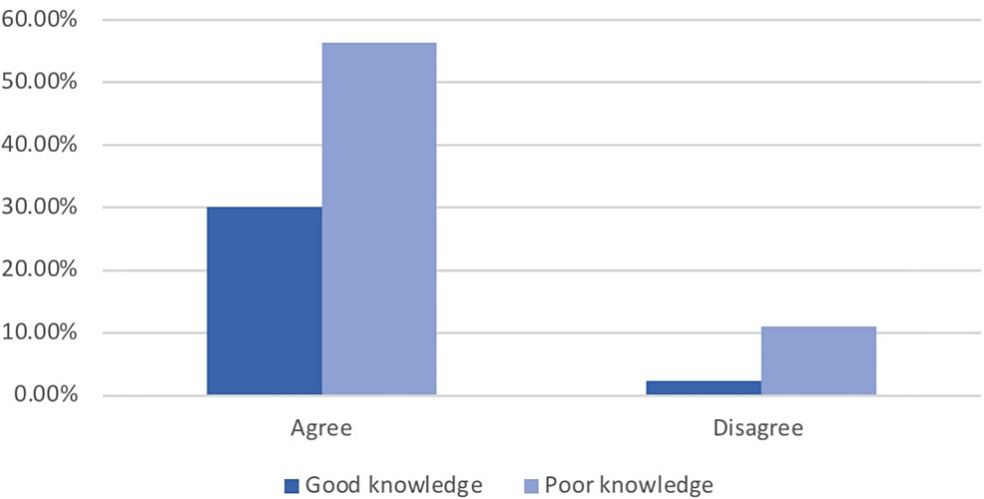
Knowledge regarding EDCs' nature and the level of agreement about their effects on the ability to have children (fertility) EDC: endocrine-disrupting chemical

The knowledge of participants was tested by asking if they agreed with the fact that hormonal disruptors may affect their reproductive health. Most of the participants agreed that there are environmental factors that may affect the menstrual cycle duration and intensity as well as fertility, even though they had poor knowledge about the nature of those products (Figures 1, 2).

A significant association was found between the participants' knowledge and their level of agreement based on the chi-square test (p=0.012, and 0.007, respectively) (Table 2).

**Table 2:** The association between the knowledge level and the usage of different endocrinal disruptors *The chi-square statistic is significant at 0.05 level

		Knowledge
Do you agree that there are environmental factors that may affect menstrual cycle duration and intensity?	Chi-square	6.287
	df	1
	Sig.	0.012^*^
Do you agree that there are environmental factors that may affect the ability to have children (fertility)?	Chi-square	7.202
	df	1
	Sig.	0.007^*^
Did you use soya-containing products for a long period?	Chi-square	21.516
	df	3
	Sig.	0.000^*^
Have you ever used any other hormonal therapy?	Chi-square	14.122
	df	2
	Sig.	0.001^*^

The statistical findings from the chi-square test showed that women with poorer knowledge about sex hormone disruptors were significantly less likely to report the long-time usage of soya-containing food when compared to women with greater knowledge (p<0.001); it also showed that women with poorer knowledge were significantly less likely to report the usage of hormonal therapies when compared to women with greater knowledge (p<0.001). These findings indicate that better knowledge regarding the nature of hormonal disruptors is not associated with a decrease in the usage of soya-containing products despite the fact that the participants are aware of the nature of these products. The study also observed no significant relationship between knowledge about sex hormone disruptors and various parameters such as age, place of residence, and social and educational status. A statement provided in the knowledge assessment (Figures 1, 2) indicated relatively good knowledge about the impact of EDCs but poor knowledge about the nature of these products and their usage.

## Discussion

EDCs have been gaining attention as they cause developmental and reproductive anomalies [8]. They also hurt the offspring and subsequent generations [9]. This is the first study of its kind to be conducted among Saudi women to assess their awareness regarding sex hormone disruptors.

In this study, the age of females ranged from 15 to 45 years, and most of them (70.7%) reported regular menstruation duration. More than one-half reported 13-15 years as the age of menarche. This information indicated that most females had normal menstruation and reached puberty at the normal age range. This reflects no problems and no disruption of sex hormones among those females. A previous study conducted on females in Pakistan showed that the large majority of females had regular periods, even those of older age. Also, the age of menarche for 43.93% of respondents was 13-14 years. Additionally, the large majority denied using hormonal replacement therapy (89.5%) [7]. The findings of this previous study are in line with ours.

Phytoestrogens are secondary plant metabolites that are metabolized to chemicals with weak estrogen activity. They may interact with estrogen receptors and result in effects similar to those of EDCs. Phytoestrogens can be classified into different families, including isoflavones. Soybeans are the most common source of isoflavones in food [10].

In this study, the utilization of different products that can affect and disrupt sex hormones was investigated. A small proportion of females reported that they used soya-containing products for a long time, but more than one-half denied using other products such as lavender and jasmine oils, and soya seeds or their derivatives. Almost one-third of females reported using oatmeal. Additionally, more than half denied using contraceptive pills, whereas only 33.6% reported using these pills; this relates to those women who find the pills a more appropriate contraception method. However, the reason for using contraceptive pills was not investigated. We found some significant statistical differences in the knowledge and the usage of soya-containing foods and hormonal therapy. The findings indicate that a good knowledge regarding the nature of these products was not associated with a decrease in their intake. 

On the other hand, on assessing the knowledge of females regarding the impact of EDC, there was a significantly large number of females with poor knowledge regarding the environmental factors that can affect their menstruation duration and intensity. Also, there was a significant correlation between the poor knowledge of females and their level of agreement with the fact that environmental factors may affect fertility.

Other studies on this subject investigated different aspects compared to what we focused on; however, we still found it worthwhile to compare the final findings of such studies with ours. One study revealed that the awareness regarding xenoestrogens among females in Pakistan was insufficient. However, that study investigated the administration of different products and foods [7]. A cross-sectional study conducted on mothers in Jeddah, Saudi Arabia, showed that only 27.6% had received information regarding xenoestrogen. Xenoestrogens are EDCs that are capable of changing and interfering with the natural actions of endogenous hormones. Mothers had limited knowledge of xenoestrogen, and their level of education significantly affected their level of knowledge [11]. In our study, education level, age, social status, and place of residence had no impact on the level of knowledge among females.

In a study from Malaysia conducted on 15 university students regarding the use of Plastic-Type Food Contact Materials (PTFCMs), it was found that the majority of students (84%) had a low level of knowledge regarding EDCs. It should be noted that the study focused on the use of PTFCMs such as canned food and bottled mineral water [8]. However, it can be still concluded that the knowledge regarding EDCs is generally inadequate among women regardless of the nature of the study population, the country where the study was conducted, and the products investigated.

In a qualitative study conducted to assess public awareness, it was reported that public awareness regarding EDCs, their sources, and associated health effects was low. This was attributed to the lack of attention EDCs get from the mainstream media, the lack of awareness programs in schools, and the lack of information from health professionals [12]. Another study that was also conducted in Jeddah, Saudi Arabia, reported a significant association between the usage of children's toys (66.3%) and the age of breast appearance; this study considered all essential factors related to puberty onset, such as height, weight, BMI, and nutritional status [13].

In a study conducted in France, 15 girls were selected and interviewed along with their parents, and asked about exposure to xenoestrogen products; of them, nine girls reported being exposed to such products. Further investigations revealed an increased level of estrogenic bioactivity in their serum, suggesting that it can be related to their exposure to estrogen disruptors. This study measured the serum levels of plasma estradiol, follicle-stimulating hormone, and luteinizing hormone [14]. The increased use of products containing endocrine disruptors, like plastic water bottles, women's cosmetics, and food preservatives impaired normal pubertal development. Therefore, exposure to these products for long periods may increase breast cancer incidence [15]. 

An Italian study in 2012 that involved 17 females with early pubertal signs of synthetic estrogen (mycoestrogen zearalenone) found that six out of the total 17 participants had elevated levels of this product without it having any significant effect on their early puberty. However, the study concerned herbicides as an environmental factor linked to elevated levels of estrogen [16].

Our study has a few limitations, primarily its relatively small sample size and the fact that the study was conducted in a small geographical area; hence it is difficult to generalize our results to other areas or populations. Also, in our descriptive study, we did not account for potential confounders, which could impact the interpretation of the findings.

## Conclusions

Females in our study had good knowledge about the nature and usage of EDCs but poor knowledge regarding their impact. The knowledge of females was associated with their behavior regarding the use of such products. Also, their knowledge affected their perceptions regarding the impact of environmental factors on menstruation and fertility.
